# Predicted Effects of Gypsy Moth Defoliation and Climate Change on Forest Carbon Dynamics in the New Jersey Pine Barrens

**DOI:** 10.1371/journal.pone.0102531

**Published:** 2014-08-13

**Authors:** Alec M. Kretchun, Robert M. Scheller, Melissa S. Lucash, Kenneth L. Clark, John Hom, Steve Van Tuyl

**Affiliations:** 1 Portland State University, Department of Environmental Science and Management, Portland, Oregon, United States of America; 2 Silas Little Experimental Forest, USDA Forest Service, New Lisbon, New Jersey, United States of America; 3 United States Department of Agriculture Forest Service, Newtown Square, Pennsylvania, United States of America; Virginia Commonwealth Univ, United States of America

## Abstract

Disturbance regimes within temperate forests can significantly impact carbon cycling. Additionally, projected climate change in combination with multiple, interacting disturbance effects may disrupt the capacity of forests to act as carbon sinks at large spatial and temporal scales. We used a spatially explicit forest succession and disturbance model, LANDIS-II, to model the effects of climate change, gypsy moth (*Lymantria dispar* L.) defoliation, and wildfire on the C dynamics of the forests of the New Jersey Pine Barrens over the next century. Climate scenarios were simulated using current climate conditions (baseline), as well as a high emissions scenario (HadCM3 A2 emissions scenario). Our results suggest that long-term changes in C cycling will be driven more by climate change than by fire or gypsy moths over the next century. We also found that simulated disturbances will affect species composition more than tree growth or C sequestration rates at the landscape level. Projected changes in tree species biomass indicate a potential increase in oaks with climate change and gypsy moth defoliation over the course of the 100-year simulation, exacerbating current successional trends towards increased oak abundance. Our research suggests that defoliation under climate change may play a critical role in increasing the variability of tree growth rates and in determining landscape species composition over the next 100 years.

## Introduction

Globally, forests are responsible for 2.4±04 Pg of carbon (C) storage, an amount equivalent to ∼30% of annual anthropogenic carbon emissions [1]. The temperate forests of the United States in particular are estimated to have increased their carbon uptake by up to 33% from the 1990s to the 2000s [1]. There is some uncertainty as to whether forest growth can continue to offset global CO_2_ emissions in the coming century [2]. Broadly speaking, forests in the United States are still recovering from post-settlement (1700-1935) carbon losses, and this recovery is expected to continue, though disturbance and future climate will likely be huge determinants [3].

Disturbance regimes within forests can have significant impacts on carbon storage and cycling. ‘Discrete’ disturbances are usually short-lived, often severe events that can have significant impacts on C fluxes (e.g. annual growth) and reservoirs (e.g. woody plant tissue)[4]. Wildfire and harvesting are two such disturbances – though they often last no more than days or weeks, their impacts can be substantial [5]. In contrast, gypsy moth outbreaks are seasonal events which can persist for several years across a region [6]. Their direct effects, such as seasonal loss of leaf area or increased moisture stress, impact ecosystem function and structure, particularly in forests of long-lived trees [7]–[9].

Climate change can be thought of as a unique, long-term disturbance in itself, or alternatively, as a driver of forest change. Climate change may alter forest structure and function in many ways, causing range shifts, influencing growth patterns [10], [11], and ultimately altering successional trends in forests. However, it remains uncertain whether and to what degree disturbance regimes will mitigate or exacerbate the effects of climate change. In many cases, a degree of synergism between climate change and disturbance regimes is expected. For example, warming temperatures may allow insects to expand into previously climatically-restricted geographic areas, which could change tree species composition and carbon sequestration potential [8], [12]. In other cases, the outcomes of one disturbance might partially offset the other. Increased growth or a longer growing season caused by warming temperatures could help mitigate some carbon loss from increased disturbance under a changing climate, though such mitigation might be lessened with the possibility of increases in ecosystem respiration [11], [13].

Because of the complexity of the interactions, understanding what processes are ultimately responsible for driving forest change is challenging. Furthermore, systems can be driven by emergent behaviors of a combination of processes, which may not become evident unless examined at decadal or centurial time scales [14]. Landscape models can help address both these issues [15], [16]. Landscape models allow individual processes to be isolated such that interactions between succession, disturbance, and climate can be decomposed to reveal how each may influence the others. These influences can then be examined at spatial and temporal scales that may not accessible by other means.

The New Jersey Pine Barrens (NJPB) is a system characterized by diverse disturbance regimes and is emblematic of risks to carbon storage in temperate forests. Previous research has suggested that the forests of the NJPB are a net C sink [7], [17]–[19], however the interactive effects of climate change and gypsy moth defoliation on C dynamics are unexplored. The area is well instrumented for analysis of forest change and carbon cycling; eddy flux data from three representative sites quantified the effects of insect defoliation and fire on NEE and evapotranspiration [7], [20].

In this study, our objectives were to investigate the landscape-scale effects of gypsy moth defoliation on the forests of the NJPB, and to understand how this disturbance interacts with wildfire and climate change. We used the LANDIS-II modeling framework to project and analyze C dynamics and species composition changes over the next 100 years. Comparison of simulations incorporating wildfire, climate change, and gypsy moth defoliation provide insight into how C dynamics may be affected by these disturbances and how major tree species may respond across the landscape. This research effort builds upon previous research in the area, including empirical data analysis [7] and simulation modeling [17], [21], [22].

## Methods

### Study Area

**Figure 1:**
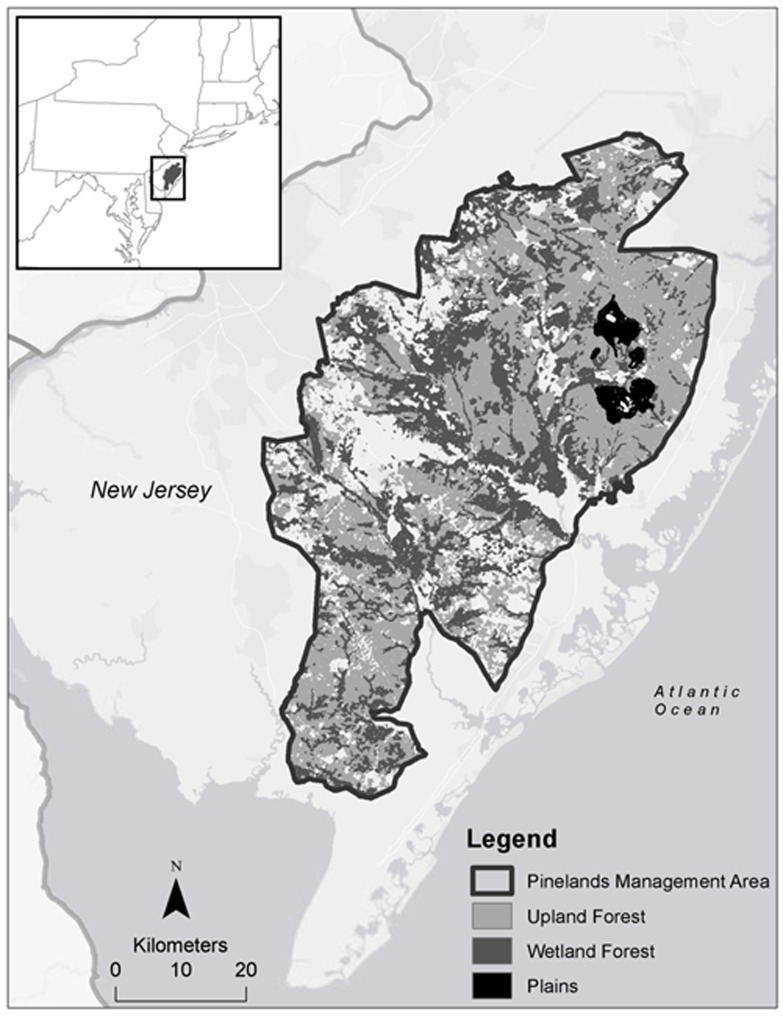
The New Jersey Pine Barrens (NJPB) and associated forest types. The Pinelands Management Area is outlined in black.

The New Jersey Pine Barrens (NJPB) is the largest contiguous forest along the Northeastern coastal plain (Fig. 1). The terrain of the NJPB is relatively homogeneous, with plains, low-angle slopes, and wetlands. The maximum elevation is 63 meters. Temperatures are cool, ranging from monthly mean temperatures of 0.3°C in January and 23.8°C in June (New Jersey State Climatologist, 1930-2009). Mean annual precipitation is 1123±182 mm. The soils are sandy, nutrient-poor, acidic, and have low cation exchange capacity [23].

From pre-settlement to ∼1940, fire played a substantial ecological role. Following the abandonment of industrial forestry in the region, a period of intense wildfires occurred, a consequence of the young age and density of the resultant stands. Fire suppression and increasing fragmentation greatly reduced the presence of wildfire on the landscape starting around 1940 [24]. Though many legacy stands remain from this period, land use change and fire suppression has greatly reduced the occurrence of large wildfires, causing a gradual shift towards a more oak- (*Quercus* sp.) dominated system [21]. Though fire occurrence has been reduced, it continues to shape forest structure.

The forests of the New Jersey Pine Barrens (NJPB) are differentiated into three primary forest types largely based on differences in soil type and disturbance history. Upland forests (58% by land area) are dominated by pitch pine (*Pinus rigida* Mill.) and several species of oak, including chestnut oak (*Quercus prinus* L.), white oak (*Q. alba* L.) and black oak (*Q. velutina*) [25]. The understory is comprised of moderate to dense shrub cover of genera *Quercus, Gallussacia, and Vaccinuim* [26]. Soils in the uplands forests are generally sandy, acidic, and nutrient-deficient [7]. Wetland forests (38% of forested land) are dominated by Atlantic white cedar (*Chamaecyparis thyoides*), moisture sensitive oaks, shortleaf (*P. echinata* Mill.) and pitch pines, and some non-oak hardwoods (e.g., blackgum, *Nyssa sylvatica*) [25]. Wetland forest soils have higher water holding capacity and this forest type is primarily associated with the lowland streams and rivers that extend from the coast and reticulate the area. The smallest forest type (<5% by land area) is the xeric pine plains. These forests are almost exclusively ‘pygmy’ pitch pine, their low productivity a consequence of the deep, well-drained, nutrient poor soils.

Gypsy moths spread into New Jersey in 1966 after their original introduction near Boston, MA in the late 1860s. Although a single year's defoliation is often not sufficient to cause substantial (>20%) mortality of overstory oaks, outbreaks typically last for several years; this cumulative defoliation is apparently the main cause of moth-attributed mortality to affected trees. Outbreaks in more xeric forests typically occur with a 5–10 year periodicity, and can last from one to three years in mixed oak-pine forests such as those in the NJPB [27]. Moth damage in the area has peaked four times since establishment: 1972, 1981,1990, and 2007 [20].

### Modeling Framework

**Figure 2:**
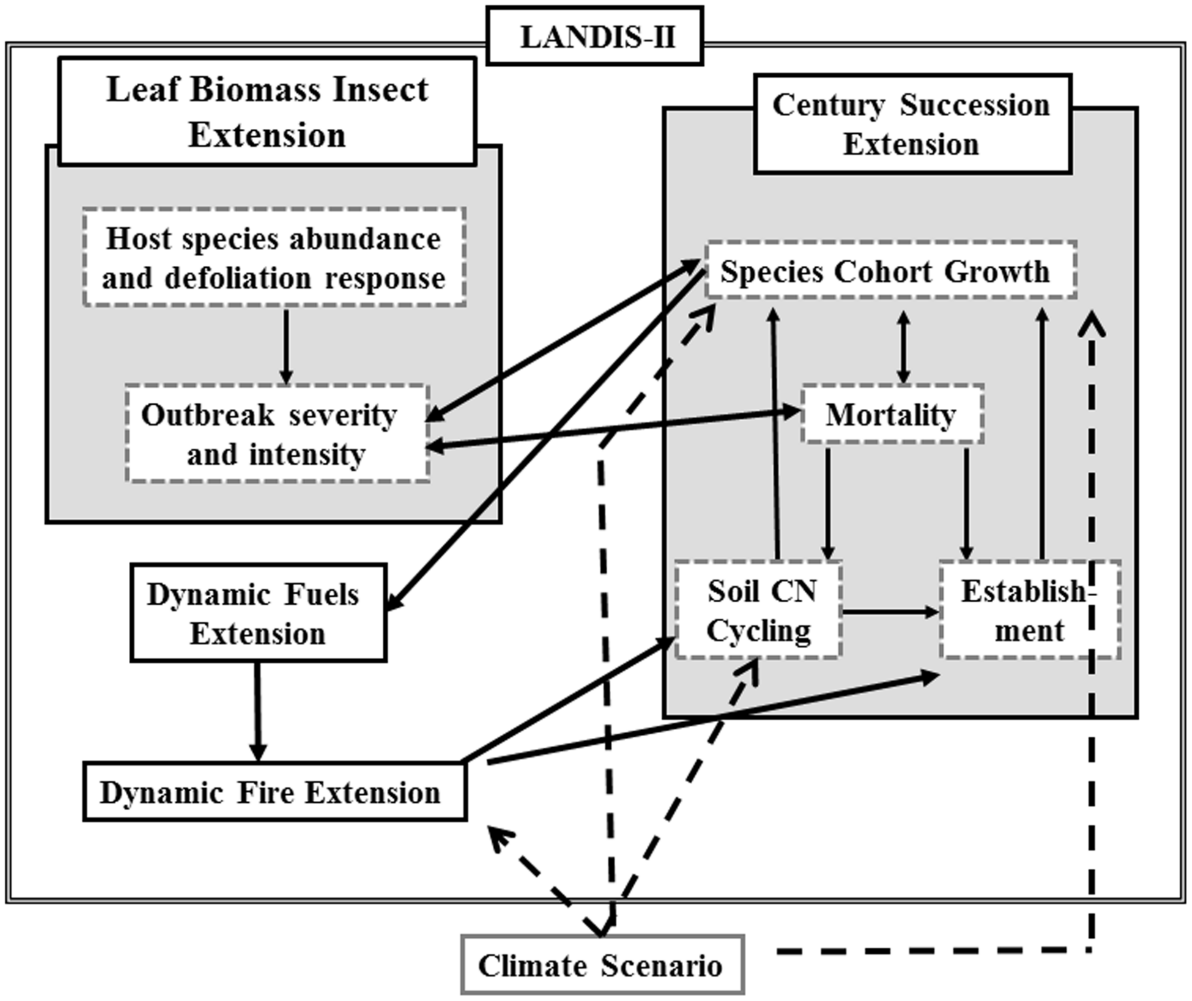
Conceptual diagram of LANDIS-II model. Arrows indicate interactions between model core, model extensions and climate scenarios.

To evaluate landscape disturbances, tree species dynamics, and C fluxes, we used the landscape forest succession and disturbance model LANDIS-II [16], [28] to model the ∼400,000 ha of southern New Jersey that falls within the Pinelands Management Area, which is managed jointly by the Pinelands Commission and the New Jersey Department of Environmental Protection. LANDIS-II simulates disturbance, management, and succession in a framework that emphasizes spatial interactions across the landscape and among processes (e.g., climate change, insects, fire, harvesting, succession, and seed dispersal) projected over decades or centuries (Fig. 2).

Rather than representing establishment and growth of individual trees, LANDIS-II bins trees into ‘cohorts’ – a grouping that organizes trees by their species and age range (e.g. pitch pine aged 11-20 years) while tracking their above ground biomass. Aggregation of trees into species-age cohorts is well suited for large study areas, as modeling individual trees would increase computation time significantly. Life history traits such as longevity and sexual maturity are required for each modeled species. At any given site, the successional dynamics of tree species are determined by their unique life history traits and their ability to establish and compete in the community in which they are found. The landscape itself is represented as a grid of interconnected cells, across which disturbances spread and dispersal occurs.

The NJPB landscape was classified into 7 different ecoregions: three upland types, three lowland types, and the xeric pine plains. Upland, lowland, and plains categorizations were determined using landcover and forest maps; the upland and lowland categories were further stratified by soil water holding capacity using the NRCS SSURGO dataset [17]. Climate and soils were assumed to be homogeneous within each ecoregion, though climate is relatively consistent among ecoregions as well [17].

With the exclusion of non-forested land use types, the total area modeled was 236,381 ha. Cell resolution was 1 ha and simulations were run for 100 years (2000–2100) at annual time steps to capture the univoltine biology of gypsy moths. Each scenario was replicated five times, the maximum allowable given our computational limits.

### Carbon and Nitrogen Simulation

The Century Succession (v3) extension for LANDIS-II (‘Century extension’) was used to simulate both above and belowground C and N dynamics and the regenerative processes of our 14 trees species [17], . Cohort biomass within the Century extension is partitioned into above- and belowground components of wood, leaves, fine roots and coarse roots. Aboveground net primary productivity (ANPP), the accumulation of aboveground biomass by tree cohorts, is determined by cohort age, longevity, and inter- and intraspecific competition for resources. Species-specific inputs determine allocation between the coarse wood and fine leaf pools. Belowground productivity is defined as a fixed percentage of aboveground productivity, with separate ratios for coarse and fine material. The Century extension tracks cohort growth at a monthly time step.

In addition to tracking cohort reproduction, growth, and mortality, the Century extension simulates changes in dead biomass (i.e., detritus) and soil organic carbon (SOC). Within the Century extension, soils are divided into fast, slow, and passive organic matter pools [30], [31]. Soil respiration is a product of climate, edaphic properties, and structural and chemical properties of detrital inputs [29]. The difference between ANPP and soil respiration is calculated by the Century extension to allow the calculation of C sequestration rates (NEE). Probability of establishment (P_est_), which describes the probability that a new cohort of a given species will become established given sufficient light and propagule presence [28], is calculated within the Century extension as a function of climate and soils.

Model calibration/validation for two critical ecosystem carbon fluxes (ANPP, NEE) against empirical data was done by matching literature values of ANPP and fitting simulated NEE values against local flux tower sites. Simulated ANPP values (450 g C m^2^) fell within the range of literature values for the region (∼440 g C m^2^) [18]. Simulated NEE was calibrated against monthly data from 2005–2007 using two eddy flux tower sites from the area, with a third used for validation. These towers were located within areas of three representative forest types in the region: oak-pine, pine-oak, and pitch pine-scrub oak [7], [20]. Model NEE estimates fit empirical flux tower data with a significant correlation of R^2^>0.6, meeting calibration criteria previously established [17]. A more detailed description of the Century extension parameterization process, along with all input parameters used, can be found elsewhere [21], [22].

### Wildfire Simulation

The Dynamic Fire system extension and Dynamic Leaf Biomass Fuels extension (known collectively as the Dynamic Fuels and Fire system, DFFS) were used to simulate fuels and fire across the landscape [32]. Wildfire ignitions are stochastically generated. The fire then spreads based upon a fire weather index (FWI) derived from climate inputs and fuel type. The Dynamic Leaf Biomass Fuels extension uses cohort characteristics (species, age-ranges, aboveground biomass) to determine fuel types for each individual cell. These fuel classifications were previously described [17]. FWI and fuel type classifications combine to determine fire intensity, which along with species-specific fire tolerances, determines cohort mortality (younger cohorts are more vulnerable) and the amount of wood and litter consumed. Model calibrations were made for current conditions; the simulation of climate change impacts on fires is an emergent property of mechanistic simulation of altered fire weather patterns [17]. Previous simulation results using LANDIS-II in the area resulted in reasonable ignition density, fire size, and acreage burned [17].

### Climate Change Data

Hadley Community Model 3 (HadCM3) climate projections were acquired from the Intergovernmental Panel on Climate Change (IPCC) data center (http://www.ipcc-data.org/). Baseline, or ‘current’, climate data was created from 30 year (1961–1990) monthly averages from the US EPA Center for Exposure Assessment Modeling (www.epa.gov/ceampubl). The A2 or ‘high emissions’ scenario was used to simulate climate change [17]. Due to ocean influences on climate, a low emission scenario (B1) did not exhibit substantial deviation from current climate (data not shown). Under the A2 scenario, temperatures steadily rose over the 100-year simulation, projecting an increase of 7.6°C in maximum summer temperatures and 5.1°C in minimum winter temperatures. Total precipitation increased slightly and became much more variable over time. The seasonality of precipitation also shifted, peaking in August at year 2000 and November at year 2100.

Climate scenarios were consistent across extensions, though each extension requires different temporal scaling of the climate stream. For the Century extension, which operated at a 5-year time step, average monthly means and standard deviations for temperature maxima, minima and precipitation were calculated from the HadCM3 A2 high emissions climate projection. The DFFS extension required daily weather and in the case of climate change scenarios, this daily weather must match the projected changes in temperature and precipitation from the HadCM3 model. Downscaling of the HadCM3 monthly climate projections was conducted using a stochastic weather generator, LARS-WG to generate daily maximum and minimum temperatures and precipitation based on the HadCM3 A2 scenario [22]. This is done by combining a reference set of daily temperature and precipitation values (collected at Atlantic City, NJ) with the HadCM3 monthly projections within LARS-WG.

### Gypsy Moth Simulation, Parameterization, and Calibration

The Leaf Biomass Insect (v1.0) extension was used to simulate landscape level effects on forest growth and mortality due to outbreaks of gypsy moths, a defoliating insect [33]. This extension stochastically introduces defoliation events uniquely parameterized for individual insect species. Temporal aspects of outbreaks are defined by specifying the duration and time between individual outbreaks. Outbreak spatial characteristics (patch size, within-patch variability, among-patch disturbance spread) are also simulated. At the cell level, outbreak spread is dependent upon adjacent defoliation levels and tree species composition.

Defoliation within the extension is defined as the amount of foliage removed from the canopy and transferred to the forest floor as unconsumed leaf matter and insect frass, resulting in reduced ANPP and possible mortality. Chemical composition of both removed foliage and insect frass is distinguished from senescent leaves within the internal nutrient cycling dynamics of the Century extension. For example, unconsumed litter that falls to the forest floor typically has much higher N content and decomposes more rapidly than litter deposited later in the year. Also, gypsy moth frass is also more readily decomposed than leaf litter. If an outbreak persists over many years, the decline in ANPP and likelihood of tree mortality increase. These two tree responses (ANPP reduction and mortality) are unique to each tree species, and operate based on ‘cumulative defoliation’ which is defined as the sum of defoliation over all the years an outbreak persists. This cumulative defoliation is then translated into percentage of cohorts removed by user defined relationships. Species growth reduction and mortality curves for gypsy moth were previously determined for all functional groups of trees within the NJPB using empirical defoliation data from mixed oak/conifer stands in Maryland [33]. These functions (growth reduction and mortality) are defined by a slope and intercept. Growth reductions were negative linear relationships for most tree species, while mortality curves are defined by exponential relationships (see File S1). Actual defoliation is determined by neighborhood defoliation intensity distributions and tree species susceptibility values. Previously developed neighborhood defoliation intensity distributions were implemented in this project.

The Leaf Biomass Insect extension was calibrated using a combination of empirical data and previous research. Defoliation data in the form of aerial flyover surveys was acquired from the US Forest Service Forest Health Monitoring program site [34]. Maps and associated attribute tables contain several aspects of forest condition, one of which is observed pest and disease damage. Where present, this damage was categorized by forest composition type, damage type (e.g. defoliation, dieback, etc.), severity (categorical), and likely agent. These maps, drawn by hand with geospatial reference aids, were digitized into files that contain all documented information on forest condition for southern New Jersey.

Defoliation patches were determined by selecting all damage attributed to gypsy moths; two categorical severity classes were present: low and high. High severity damage was classified as >75% reduction in deciduous canopy foliage, following NJ state protocols. For this project, only areas of high severity were chosen to calibrate initial outbreak patches and the damage agent classification.

Temporal characteristics of gypsy moth outbreaks as well as host species preferences were parameterized from local empirical data. Outbreak patch size distributions were determined with the R v.2.13 statistical software package [35]. Growth reduction and mortality response curve parameters were taken from previous work [33]. Total defoliated sites in the initial year of simulated outbreaks were matched with total defoliation recorded in the flyover maps for the year 2007, the first year of the most recent outbreak in the area. Because of the binary nature of the empirical defoliation data, matching total defoliation area in the initial year of actual outbreaks with total defoliation area in the initial year of simulated outbreaks, was the most practical method for calibrating total area defoliated. Calibration involved iteratively changing the ‘Initial area calibrator’ parameter until mean simulated landscape defoliation fell within mean observed landscape outbreak area. See File S1 (Insect Extension Parameters) for complete list of input parameters.

## Results

Calibration of the Leaf Insect Biomass extension based on total defoliated area was generally successful. Total empirical defoliation across the landscape for 2007 was 63,886 ha. Average defoliated sites (1 ha) for the initial year of simulated outbreaks across simulation replicates was 64,663±6,326 ha. Patches were clustered in both empirical and modeled outbreaks. Patches sizes followed a Gaussian distribution (λ = 156.86, k = .57), with small patches (<20ha) much more frequent than large ones (>200ha) in both empirical and modeled outbreaks. The largest empirically observed contiguous patch (year 2007) in the study area was 22,861ha, while the smallest was 1ha. Maximum modeled contiguous patches were 20,148±1460ha, Minimum outbreak patch size was restricted to 1ha, as this was the model resolution.

Simulated fire and gypsy moth outbreaks were spatially disparate. Over the course of a single model replicate, fire occurred on an average of 0.08% of sites undergoing an active gypsy moth outbreak. This trend was consistent across both current climate and the high emissions climate scenario. Differences in fire severity in the presence of gypsy moth defoliation when present were small and showed no consistent pattern across ecoregions. Finally, carbon volatilized by wildfire was not significantly different between ecoregions or gypsy moth scenarios.

**Figure 3:**
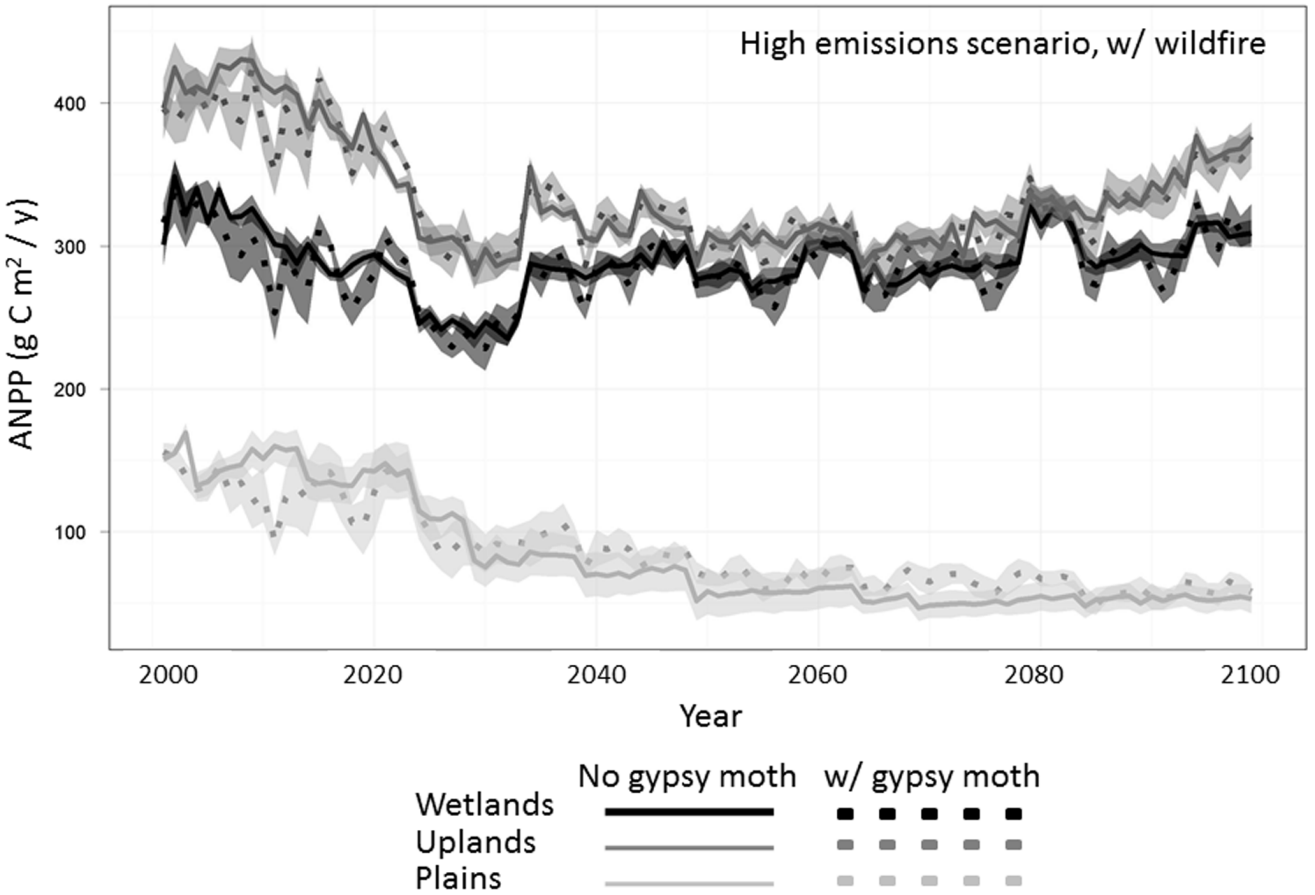
Aboveground net primary production (ANPP) in the three modeled ecoregion types under A2 (high emissions) climate scenario. Scenarios which included gypsy moth defoliation are seen in the dotted lines. Ribbons surrounding each line represent the standard error, based on five model replicates.

Simulated ANPP sharply declined during years of defoliation under a high emissions climate scenario (A2) and simulated wildfire (Fig. 3). These declines were most apparent in the upland and wetland forests. However reductions were temporary and growth rebounded quickly, causing a short-term spike in ANPP. Ultimately, gypsy moths did not significantly alter the magnitude but greatly increased variability of ANPP in the uplands and wetlands by the end of the simulation. In the plains, gypsy moths marginally increased ANPP beginning in simulation year 30 under climate change.

Carbon sequestration rates exhibited similar trends to ANPP, showing no major changes in landscape-level NEE due to gypsy moths over the course of the simulation (data not shown). During modeled outbreaks, individual sites became temporary C sources, but the landscape as a whole remained a net C sink throughout the simulation period.

**Figure 4:**
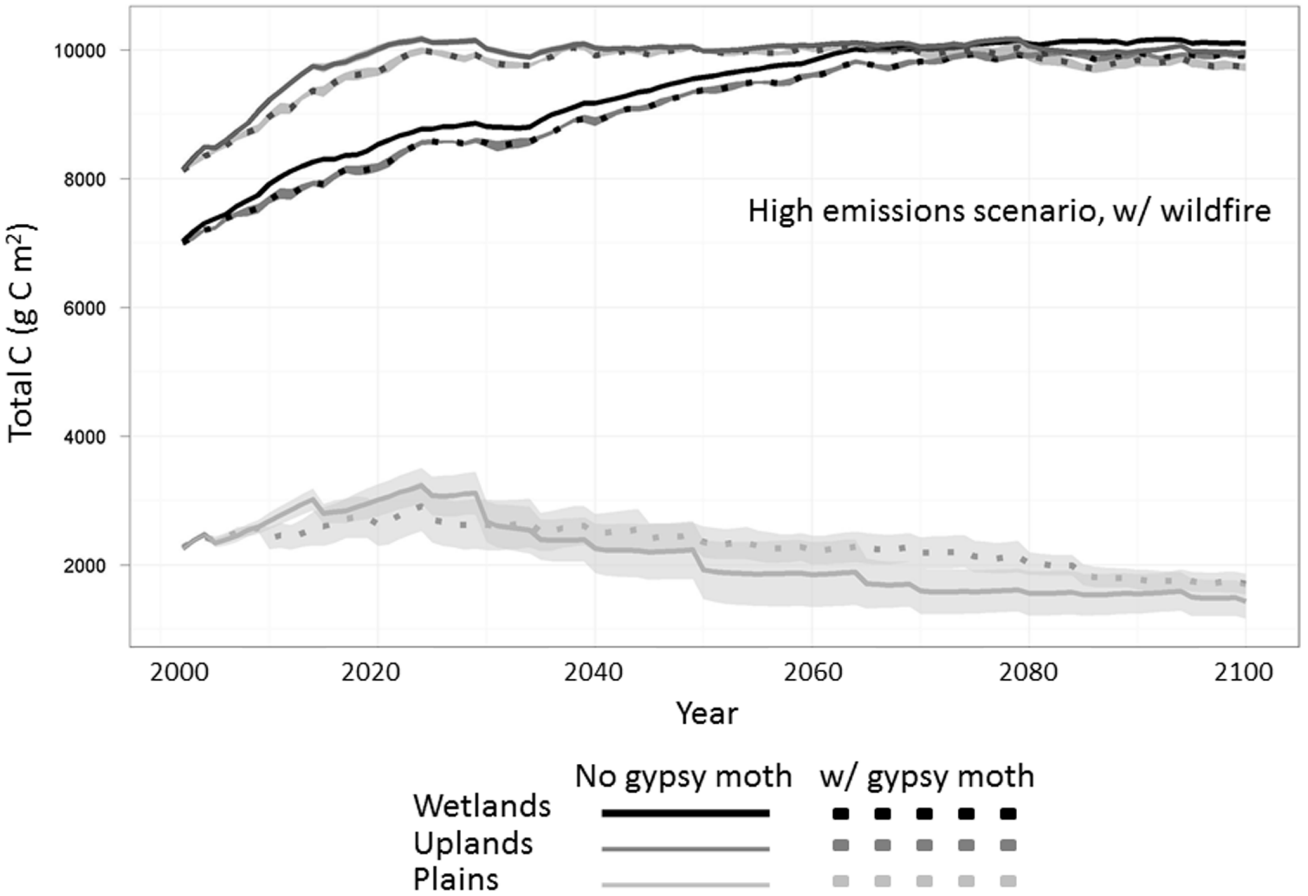
Total carbon under A2 (high emissions) climate scenario for all modeled ecoregion types. Reductions in the uplands and wetlands are a consequence of gypsy moth defoliation (dotted lines). Total carbon inthe plains, represented in light grey, did not significantly change over the 100-year scenario.

Gypsy moths did not drastically shift change the temporal pattern of most major C pools, but did affect the magnitude of total carbon. Under climate change, gypsy moth presence reduced total C in the upland and wetland ecoregions (Fig. 4). In the plains, total C was slightly higher in the presence of gypsy moths, though this increase was within the bounds of our 5-replicate variability.

**Figure 5:**
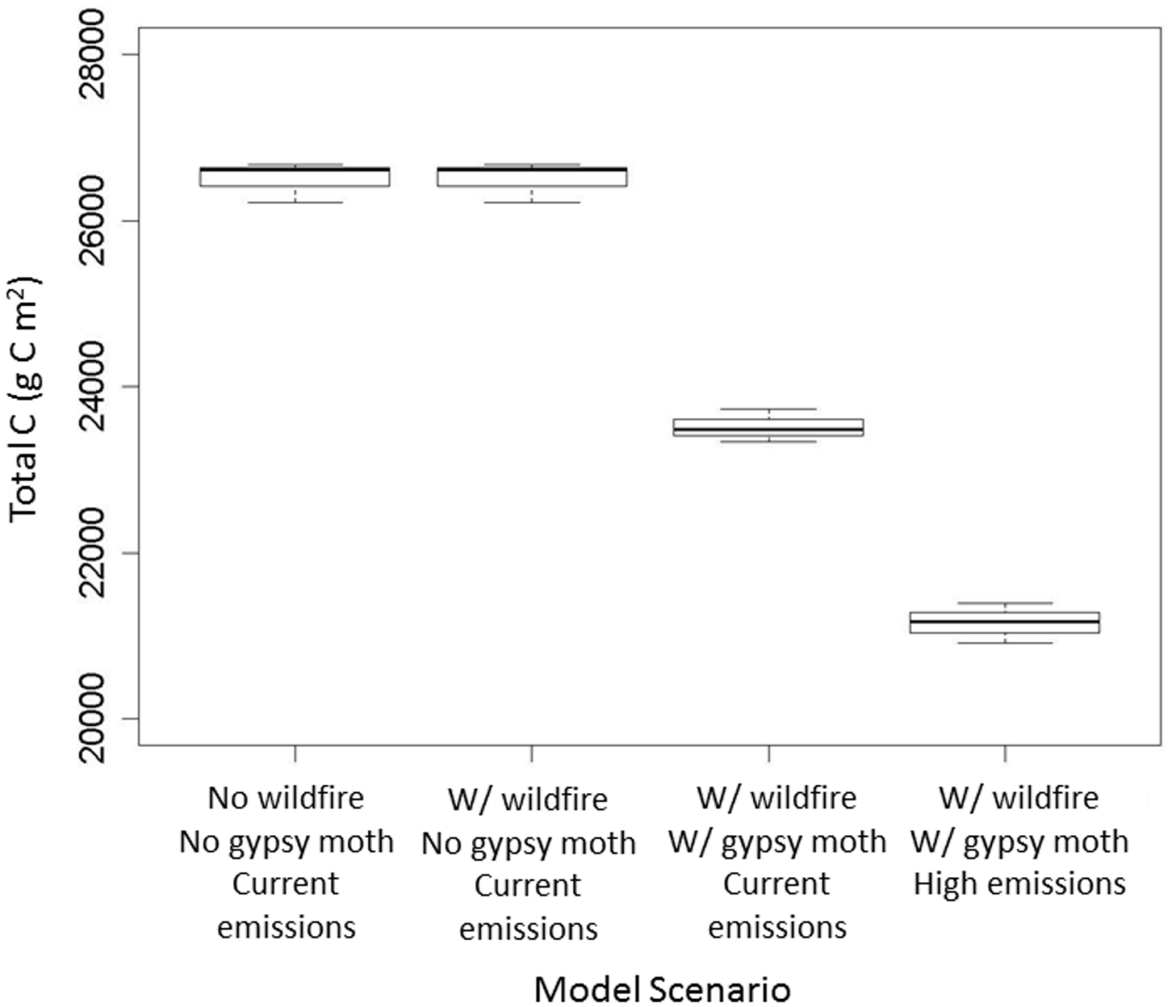
Total ecosystem carbon in four different model scenarios at simulation year 100. Scenarios 1–3 use baseline (current) climate, while the fourth uses the A2 (high emissions) scenario. ‘w/GM’ implies inclusion of gypsy moth outbreaks in the scenario. Confidence intervals represent standard errors based on 5 model replicates. Total carbon, was lowest when all three disturbances were operating on the landscape. Simulated wildfire had little impact on total C.

In addition to examining the effects of defoliation under simulated wildfire and climate change (Figures 3 and 4), we also looked at the relative magnitude of each individual disturbance and their interactive effects on total C (sum of all above- and belowground C pools). When comparing the effects of different disturbances on total ecosystem carbon, climate change had the largest effect on total C, followed by gypsy moth presence (Fig. 5).

**Figure 6:**
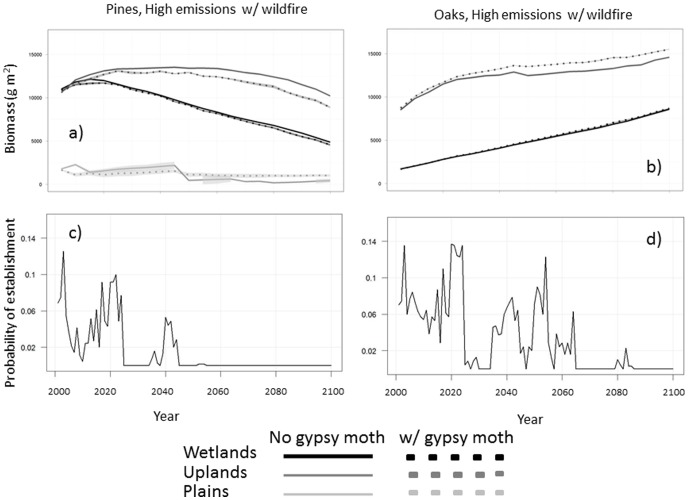
Aboveground biomass (g m^−2^) for ‘Oak’ and ‘Pine’ functional groups over the course of the 100-year simulations. Plots *a* and *b* are comparisons of these groups under high emissions (A2) climate; Dotted lines represent scenarios with gypsy moth defoliation present. Ribbons surrounding each line are standard errors, based on five model replicates. Plots *c* and *d* show landscape probability of establishment for pine and oak functional groups under high emissions climate.

Functional tree species types were differentially affected by gypsy moth defoliation and the effects varied by ecoregion. Under high emissions climate, the pines decreased in biomass in the upland and wetland ecoregions with gypsy moth defoliation (Fig. 6a). Oak biomass significantly increased with gypsy moth defoliation in the upland and wetland ecoregions under baseline climate at simulation year 2100 (Fig. 6b). Under climate change, this increase in oaks was seen in the uplands, but was not present in the wetlands or plains (Fig. 6b).

“Probability of establishment (P_est_) for both oak and pine functional groups fell throughout the 100 year simulations (Fig 6c and 6d). P_est_ in the oak group fell at a slower rate than that of the pines, resulting in significantly higher establishment probability by the middle of the 100 year simulations. Table 1 compares the average difference in aerially-weighted biomass at simulation year 100 for four scenario pairs: two functional tree types under two climate scenarios with and without gypsy moth defoliation. The oak functional group showed statistically significant increases with gypsy moth defoliation regardless of climate scenario. The pine group showed decreases under both climate scenarios with gypsy moth defoliation; however the decrease was much greater and proved statistically significant under high emissions climate.

**Table 1:** Difference in biomass of functional species groups w/gypsy moth defoliation.

Functional group	Scenario comparison	Biomass difference (g C m^2^)
Oaks	Baseline emissions, baseline emissions w/GM	+283.69*
	High emissions, high emissions w/GM	+191.33*
Pines	Baseline emissions, baseline emissions w/GM	−76.6
	High emissions, high emissions w/GM	−232.70*

Legend/footnotes: Difference in area-weighted biomass of two major functional groups for 4 scenario comparisons at simulation year 100 across the landscape. GM represents inclusion of gypsy moth in the scenario. Positive values indicate higher values in the second listed scenario, negative larger in the first scenario. * denotes significance at *p* = 0.05 as determined by pairwise Tukey's HSD.

## Discussion

Our research objective was to understand the interactive effects of gypsy moth and climate change under a fire regime on the forests of the NJPB with respect to landscape C pools and fluxes, as well as forest species composition and distribution. The spatially explicit and dynamic nature of our simulations allowed for analysis of these effects both across the entire landscape and at the one hectare scale. Our results suggest that long-term patterns in total C will be more affected by climate change than by fire or gypsy moth defoliation over the next century. When simulated gypsy moth outbreaks occurred, defoliation had a large effect on C sequestration rates (i.e. NEE) at the local scale, but not at the landscape scale. At the landscape level, gypsy moth had minor effects on ANPP and NEE, although substantially altered species composition in favor of oaks to the detriment of pines.

One of the primary effects of gypsy moth defoliation is an inhibition of tree growth via a decline in leaf biomass [7], [9], [36]. Such inhibition is species-specific in its magnitude, but can be cumulatively substantial at the landscape scale. This growth reduction was captured in our simulations, which generated short-term declines in ANPP during gypsy moth outbreaks in the uplands and wetlands (Fig. 3). Despite this reduction, ANPP rebounded to previous levels within 1–2 years, suggesting that defoliation of gypsy moths may have little impact on growth rates at the landscape-level. Though few studies have looked at ANPP specifically in response to defoliation, long-term data from tree rings in the region suggest that radial growth in affected trees is reduced by the previous year's defoliation [37]. Likewise, projected outbreaks of spruce budworm (*Choristoneura spp*) in Eastern Canadian forests also result in reduced net primary productivity, consequently reducing carbon sequestration [38].

The transient nature of growth reduction emphasizes the need for careful consideration of temporal and spatial scale when simulating forest disturbances. At the landscape level, defoliated sites represented an average 27% of the modeled study extent during individual major gypsy moth outbreaks. This was similar to estimates of the most recent major defoliation event occurring in 2005–2008 (20.1% of upland forests damaged) [7], though matching the complexity and heterogeneity of tree damage within outbreak patches is difficult due to the categorical nature of the aerial survey maps used to calibrate gypsy moth patch size. Defoliation in our scenarios caused small, short-term changes in ANPP during an outbreak, but the overall magnitude and trajectory of productivity were not significantly altered over the long term at the landscape scale (Fig. 3). Additionally, during modeled outbreaks many individual sites became temporary C sources; however the potential of these forests across the landscape to act as a C sink was not wholly compromised. Medvigy et al [39] also noted the discrepancy between regionally averaged defoliation effects and effects within defoliation patches.

Total carbon shows modest decreases with gypsy moth defoliation, as leaf biomass represents a relatively small proportion of the carbon stored in forests (Fig. 4) [7]. Though a spin-up phase is incorporated into the model, disturbances are not included in this period and thus initial decreases in ANPP and modeled carbon stocks (Figs. 3, 4, and 5) are likely due to the ‘introduction’ of gypsy moth at year 2010 of our simulations. This may account for the large initial decline in ANPP, though reductions continue throughout the simulated century.

Following defoliation events, tree species that are less susceptible to gypsy moth defoliation (e.g. pines) may have a competitive advantage over preferentially selected host species (e.g. oaks), perhaps shifting stand composition towards the former over long periods of time [40], [41]. Our results instead projected a perpetuation of the current compositional trend towards more oak domination in the NJPB [21], [42]. During large outbreaks, gypsy moths are relatively indiscriminate in their host choice, and though oaks are generally more preferred hosts, many oak species are more resilient or resistant to defoliation-caused mortality than conifers [43]. Typically, only latter instar gypsy moth larvae will defoliate conifers and only when gypsy moth densities are particularly high [37]. Our simulations of mortality in response to defoliation made conifers more susceptible to mortality during outbreaks [33]. Similarly, Lovett et al [43] observed differing mortality rates in mixed oak/conifer stands were responsible for propagation of oaks after gypsy moth outbreaks.

There is some uncertainty to the long-term successional trends of defoliated forests, particularly when focusing on forests of mixed oak-conifer stands [43]. Such uncertainty is compounded in our study, which considers gypsy moth impacts, but also projected climate change influences as well as fire. Although prescribed burns and wildfire do occur on the landscape [17], [36], they more frequently occur in the ‘sand plains’ where gypsy moth outbreaks are not prevalent. As such the interaction between these two disturbances was limited. Field measurements from affected oak-dominated stands in the region have shown increased growth increments in pines and a reduction in live oak biomass immediately following an outbreak, as well as release of understory shrubs [37]. The discrepancy between field observations and model results is due to 1) differences in mortality rates between oaks and pines and the resulting long-term effects of regular outbreaks on species composition, and 2) declining pine establishment caused by changing climate over one hundred years. Though establishment in both pitch pine and white oak fell throughout the course of the climate change scenario, the decline in establishment of pitch pine was more severe [22] (Fig 6). Therefore at the scale of our study, higher pine mortality rates during outbreaks, along with projected declines in pine establishment, were stronger than the differences in host preference. Although some previous studies have concluded the opposite (e.g.[44]), work by Foster [33], which we used to parameterize the gypsy moth outbreaks in this study, found that pine mortality exceeded oak mortality in mixed oak-conifer stands. These effects exacerbate the current trend towards more oak-domination, a consequence primarily of succession and fire exclusion [21], [42] and strongly suggest that long-term climate effects are additive with shorter-term gypsy moth outbreaks (Table 1).

For all modeled processes we are limited, as always, by the availability of data used for parameterization. Many parameters used in the succession and disturbance extensions, though derived from empirical data, are themselves uncertain. Data used in establishing growth declines and mortality rates in response to gypsy moth were empirically derived, though the study sites were primarily in Maryland. Soil carbon estimates used for this study are based on limited sampling extrapolated across a broad scale, a weakness previously noted in Scheller et al. [17]. Confidence in many species’ life history attributes and disturbance parameters is high, primarily because the NJPB has been an extensively studied system. However, uncertainty in the GCM climate projections used is extremely high, a fact underscored by the sheer number of available projections. Also, the direct impacts of temperature on gypsy moth development and dispersal are not explicitly modeled within this study. Instead, the primary interaction between gypsy moth and climate is expressed through host distribution and abundance, which is considered a determining factor of outbreak severity [45].

Though the cumulative uncertainty in parameters is not trivial, we believe that our results can be used to reliably infer the magnitude and direction of relationships among processes and offer insight into the interactions of global change processes that would be impossible with any other approach.

Though interactions among many important processes were investigated, some model uncertainty comes from the necessary exclusion of several others, namely continuing fragmentation [42] and the recently introduced southern pine beetle (*Dendroctonus frontalis* Zimmermann). Southern pine beetle infestation risk is expected to increase with climate change [46], and with the high pine mortality rates associated with these infestations, this newly introduced forest pest will likely have significant impacts on the NJPB. Atmospheric influences on tree growth and development related to climate change, including possible CO_2_ fertilization and changing O_3_ levels, were not included in our climate change projections [19]. Previous research suggests that these two greenhouse gases affect tree growth in opposing ways, though these effects are not reflected in our model.

In summary, our results suggest that gypsy moth defoliation may not affect landscape-level ANPP or C sequestration rates, but rather accelerate the shift from an oak-pine/pine-oak system to an oak-dominated one. These simulations incorporate a number of interacting forest processes (e.g. soil C and nutrient cycling, species composition), each uniquely sensitive to both disturbance and climate which are key to forecasting C dynamics at a landscape scale. The considerable variation between model replicates is due to the stochastic nature of simulated wildfire and modeled gypsy moth outbreak events. This variation draws attention to the uncertainty inherent in ecological forecasting [17], [29].

## Supporting Information

File S1 **Insect Extension Parameters.**
(DOCX)
