# Supplementary material for: Predicted Effects of Gypsy Moth Defoliation and Climate Change on Forest Carbon Dynamics in the New Jersey Pine Barrens
Source: PLoS One. 2014 Aug 13;9(8):e102531. doi: 10.1371/journal.pone.0102531 (PMC4138007; doi:10.1371/journal.pone.0102531)
Supplement: File S1 — Insect Extension Parameters. (DOCX) [file pone.0102531.s001.docx]

LandisData InsectDefoliator

InsectName GypsyMoth

MeanDuration 2

StdDevDuration 1

MeanTimeBetweenOutbreaks 7

StdDevTimeBetweenOutbreaks 2

NeighborhoodSize 250 << meters

InitialPatchShapeCalibrator 1.0

InitialPatchOutbreakSensitivity 0.0024

InitialPatchDistribution Weibull <<Weibull, Beta, or Gamma

>> Patch size distribution

InitialPatchValue1 .57

InitialPatchValue2 156.86

SpeciesParameters

>> Susceptibility class by species

>> Species Susceptibility Growth Reduction Parameters Mortality Parameters

>> Class Slope Intercept Slope Intercept

>>--------------------------------------------------------------------------------

acerrubr 2 -0.8 1 .009 7 <

chamthyo 3 -1 1 .009 7

nysssylv 3 -0.15 1 .009 7

pinuechi 2 -1 1 .016 4

pinurigi 2 -1 1 .016 4

queralba 1 -0.16 1 .006 5.5

quercocc 1 -0.16 1 .006 5.5

querfalc 1 -0.16 1 .006 5.5

querprin 1 -0.16 1 .006 5.5

quervelu 1 -0.16 1 .006 5.5

liqustyr 1 -0.16 1 .006 5.5

sassalbi 1 -0.15 1 .006 5.5

querilic 2 -0.16 1 .006 5.5

quermari 1 -0.16 1 .006 5.5

Susceptibilities

>> Host Dist80 S1_80 S2_80 Dist60 S1_60 S2_60 Dist40 S1_40 S2_40 Dist20 S1_20 S2_20 Dist0 S1_0 S2_0

1 Beta 0.84 0.33 Beta 1.27 0.68 Beta 1.14 1.18 Beta 0.76 1.68 Beta 0.8 6.0

2 Beta 1.27 0.68 Beta 1.14 1.18 Beta 0.8 1.68 Beta 0.5 1.68 Beta 0.7 6.0

3 Beta 1 1 Beta 0.8 1.68 Beta 0.5 1.68 Beta 0.3 1.68 Beta 0.5 6.0

MapNames ..\..\insects/severity-{timestep}.img

LogFile ..\..\insects/log.csv
